# Electrolyte disturbance profiles and associated factors in Somali patients with heart failure

**DOI:** 10.1097/MS9.0000000000005364

**Published:** 2026-09-08

**Authors:** Said Abdirahman Ahmed, Abdullahi Mohamed Hassan Fujeyra, Isaak Mohamed Osman, Ahmed Shafie Aden, Mohamed Omar Hassan, Ahmed Elmi Abdi, Mohamud Mire Waberi, Mohamed Abdullahi Mohamud, Yahye Abukar Sabrie, Mohamed Sheikh Hassan, Rahma Yusuf Haji Mohamud, Abdijalil Abdullahi Ali, Ishak Ahmed Abdi, Abdinafic Mohamud Hussein, Usama Hassan Hussein, Kowthar Ismail Hashi, Ali Jimale Mohamed, Mehmet Zafer Aydin, Feyza Aksu, Osman Farah Dahir

**Affiliations:** a Department of Cardiology, Mogadishu Somali-Türkiye Recep Tayyip Erdoğan Training and Research Hospital, Mogadishu, Somalia; b Faculty of Medicine and Surgery, Al Hayat Medical University, Mogadishu, Somalia; c Faculty of Medicine and Surgery, Somali National university, Mogadishu, Somalia; d Department of Neurology, Mogadishu Somali-Türkiye Recep Tayyip Erdoğan Training and Research Hospital, Mogadishu, Somalia; e Department of Nursing, Mogadishu Somali-Türkiye Recep Tayyip Erdoğan Training and Research Hospital, Mogadishu, Somalia; f Department of Cardiovascular Surgery, Mogadishu Somali-Türkiye Recep Tayyip Erdoğan Training and Research Hospital, Mogadishu, Somalia; g Department of Cardiology, Kayseri City Education and Training Hospital, Kayseri, Turkey; h Department of Cardiology, T.C. Goztepe Prof. Dr. Suleyman Yalcin City Hospital, Istanbul, Turkey

**Keywords:** electrolyte disturbances, heart failure, hypokalemia, hypomagnesemia, hyponatremia

## Abstract

**Background:**

Electrolyte disturbances are frequent yet under-monitored complications of heart failure (HF), contributing to arrhythmias, prolonged hospitalization, and increased mortality. Data from Somalia remain scarce, despite the growing HF burden.

**Methods:**

A retrospective cross-sectional study was conducted among 369 adult HF patients admitted to Mogadishu Somali-Turkish Training and Research Hospital between January 2023 and January 2025. Demographic, clinical, and laboratory data were analyzed using IBM SPSS Statistics v29. Electrolyte disturbances were classified by standard cut-offs, and associations with clinical variables were tested using Chi-square and Pearson correlation analyses.

**Results:**

The mean age was 60.1 ± 15.2 years, and 43.6% were female. The mean ejection fraction was 31.3 ± 8.0% (range 10–45%), with most patients having reduced EF. Hyponatremia occurred in 38.5%, hypokalemia in 17.1%, and hypomagnesemia in 16.3%. Among affected patients, severe hyponatremia was most frequent (26.8%), while hypokalemia was largely mild (73.0%). Loop-diuretic therapy was significantly associated with hyponatremia and hypokalemia (*P* < 0.05), while electrolyte levels showed no significant correlation with age or ejection fraction (|*r*| ≤ 0.14).

**Conclusion:**

Electrolyte disturbances – particularly hyponatremia and hypokalemia – are highly prevalent among Somali HF patients. These findings highlight the need for improved electrolyte monitoring in HF management in low-resource settings. Strengthening electrolyte monitoring and improving diagnostic capacity are essential for optimizing HF management in low-resource settings.

## Introduction

Heart failure (HF) is a complex clinical syndrome characterized by structural or functional cardiac abnormalities that impair the ability of the heart to pump or fill adequately. According to the universal definition proposed by international cardiology societies, HF is defined by symptoms and signs resulting from cardiac dysfunction, together with objective evidence of structural heart disease or elevated natriuretic peptides^[^1^]^.

HIGHLIGHTS

- Hyponatremia was the most common electrolyte disturbance in Somali HF patients.
- Electrolyte abnormalities affected a substantial proportion of hospitalized HF patients.
- Sodium imbalance occurred in 38.5% of patients in this Somali cohort.
- Loop-diuretic therapy was associated with hyponatremia and hypokalemia.
- Strengthening electrolyte monitoring may improve HF management in low-resource settings.

Globally, HF represents a major public health problem, affecting approximately 64 million people worldwide^[^2^]^. The burden of HF is expected to rise further due to increasing life expectancy and improved survival from other cardiovascular diseases^[^3^]^. The lifetime risk of developing HF is estimated to be approximately 20% for both men and women. However, the epidemiology of HF differs by age and sex, with men having a higher prevalence at younger ages, while older women more frequently develop HF with preserved ejection fraction^[^4^]^.

In sub-Saharan Africa, HF represents a substantial proportion of medical hospitalizations, accounting for approximately 9–42% of admissions in general medical wards and up to one-third of admissions in cardiology units^[^5^]^. Limited diagnostic infrastructure and late presentation frequently complicate management in these settings.

Maintaining fluid and electrolyte balance is essential for normal physiological function. Electrolytes such as sodium, potassium, chloride, and magnesium play fundamental roles in cellular metabolism, neuromuscular activity, and acid-base balance^[^6^]^.

The kidneys and cardiovascular system work together to regulate these electrolytes and maintain internal homeostasis. Electrolyte abnormalities (EAs) are common among patients with HF and may arise due to neurohormonal activation, renal dysfunction, or pharmacological therapy – particularly diuretics and renin–angiotensin–aldosterone system inhibitors^[^7^]^. Sodium and potassium disturbances are particularly important in HF because they may influence treatment decisions and are associated with adverse clinical outcomes, including arrhythmias and increased mortality^[^8^]^.

Globally, the occurrence of EAs in individuals with HF varies considerably depending on population characteristics, treatment practices, and healthcare infrastructure^[^9^]^. However, evidence from Somalia remains scarce. Understanding the prevalence and patterns of electrolyte disturbances in this population is important for improving HF management in resource-limited healthcare environments.

Therefore, this study aimed to assess the prevalence and patterns of electrolyte disturbances among HF patients admitted to a tertiary hospital in Mogadishu, Somalia.

## Methodology

### Study population and data retrieval

The study included both newly diagnosed and previously known HF patients who were admitted to the Cardiology Inpatient Department between January 2023 and January 2025. A retrospective chart review was conducted, collecting data from patient medical records, including demographic details (age and gender) and laboratory parameters such as sodium, potassium, and magnesium levels.

The review also included information on medications commonly prescribed in the management of HF, such as diuretics, angiotensin receptor blockers (ARBs), angiotensin-converting enzyme inhibitors (ACEis), and mineralocorticoid receptor antagonists (e.g., spironolactone/Aldactone), as well as other relevant cardiovascular therapies. Additionally, key cardiovascular risk factors were documented, including diabetes mellitus (DM), hypertension (HTN), dyslipidemia, and a history of renal failure.

### Eligibility criteria

Participants were eligible for inclusion if they were either newly diagnosed or previously known HF patients aged 18 years or older, with a confirmed diagnosis based on clinical evaluation and echocardiographic findings documented in hospital records prior to admission (see Fig. 1). Only patients with documented echocardiographic measurements available in hospital records were included in the analysis.

**Figure 1.**
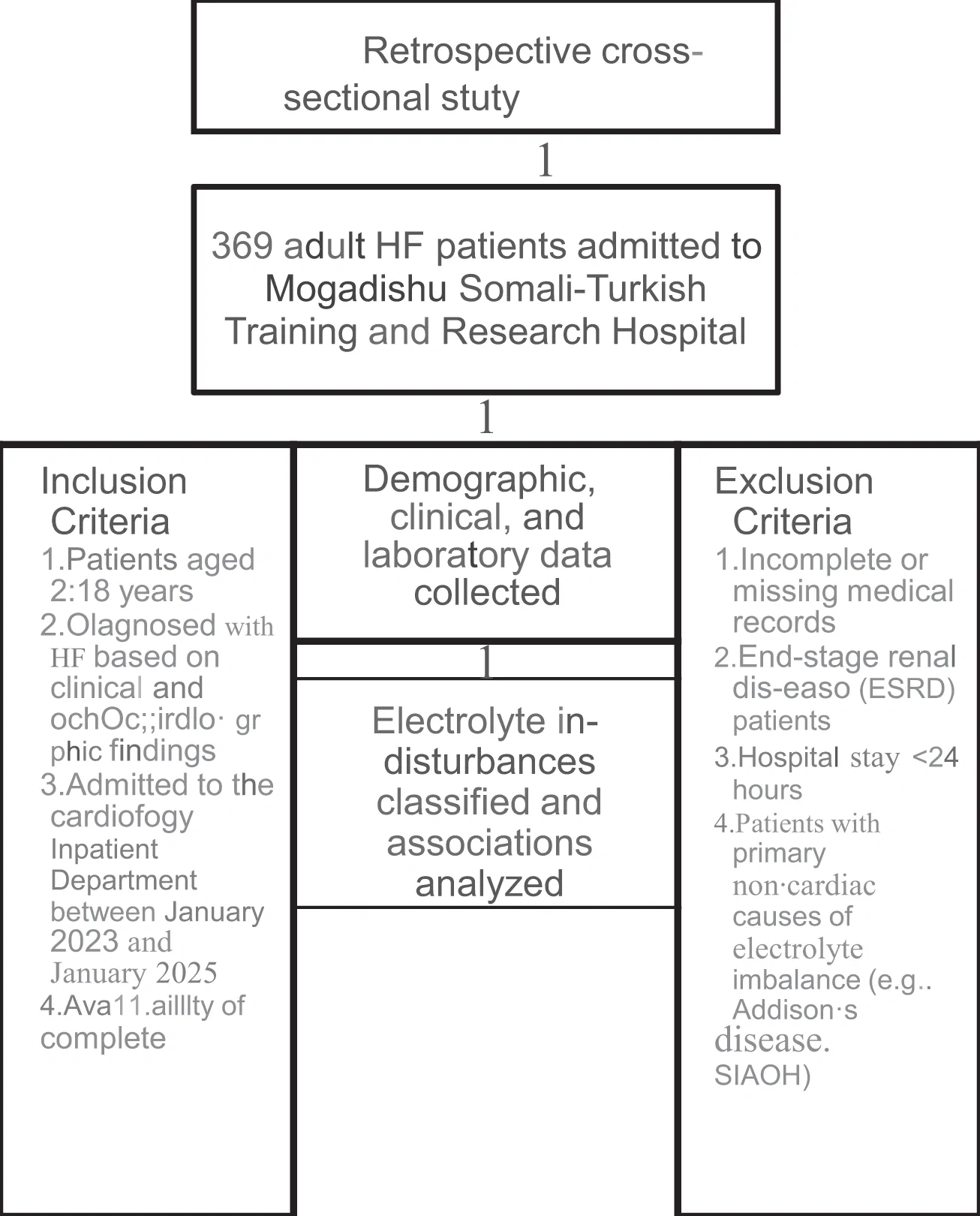
Flow chart shows eligibility criteria inclusion, and exclusion criteria.

### Statistical analysis

Data were analyzed using IBM SPSS Statistics version 29.0. Continuous variables (age, ejection fraction, electrolytes) were summarized as mean ± SD and range, while categorical variables were expressed as frequencies and percentages.

Prevalence of hyponatremia, hypokalemia, and hypomagnesemia was calculated for all available data.

Because this was a retrospective study, electrolyte measurements were not available for all patients; therefore, prevalence estimates were calculated based on the number of patients with available laboratory results for each electrolyte parameter.

Associations between categorical variables were tested using the Chi-square or Fisher’s exact test, and correlations between continuous variables were assessed using Pearson’s correlation coefficient (*r*).

All analyses were two-tailed, with *P* < 0.05 considered statistically significant. Results are presented in tables and figures, with reference lines marking clinical cut-offs.

### Ethical consideration

The study complied with the ethical principles outlined in the Declaration of Helsinki. General consent for the use of anonymized clinical data for research purposes is routinely obtained from patients upon hospital admission. Approval for the study was granted by the institutional ethics committee.

Artificial intelligence tools were used only for language editing during manuscript preparation, and all scientific interpretations and conclusions were verified by the authors in accordance with the TITAN guideline for transparent reporting of AI use in research^[^10^]^.

## Results

A total of 369 patients with HF were included. The mean age was 60.1 ± 15.2 years (range 18–105), and females constituted 43.6% of the participants. Baseline characteristics of the study population are presented in Table 1.

**Table 1:** Sample characteristics.

Metric	Value
Sample size (*n*)	369
Age, mean ± SD (years)	60.1 ± 15.2
Age range (years)	18–105
Female (%)	43.6
EF, mean ± SD (%)	31.3 ± 8.0
EF range (%)	10–45
HF duration >1 year (%)	57.5

Most patients had reduced ejection fraction, with a mean EF of 31.3 ± 8.0% (range 10–45%). The majority had long-standing HF, with 57.5% reporting a duration greater than 1 year.

### Electrolyte disturbance profile

The overall prevalence of electrolyte disturbances is summarized in Table 2. Hyponatremia (Na <135 mmol/L) was observed in 142 patients (38.5%) among those with available sodium measurements. Hypokalemia (K <3.5 mmol/L) occurred in 63 patients (17.1%) among those with available potassium measurements. Hypomagnesemia (Mg <1.7 mmol/L) was identified in 60 patients (16.3%) among those with available magnesium measurements. Distribution histograms are presented in Figure 2A–C, and the prevalence and severity of electrolyte disturbances are shown in Figure 3A–D.

**Table 2:** Electrolyte disturbance prevalence.

Disturbance	*n* (cases)	*N* available	Prevalence (%)
Hyponatremia	142	369*	38.5
Hypokalemia	63	369*	17.1
Hypomagnesemia	60	369*	16.3

^*^ Percentages were calculated based on the number of patients with available laboratory measurements for each electrolyte parameter.

**Figure 2.**
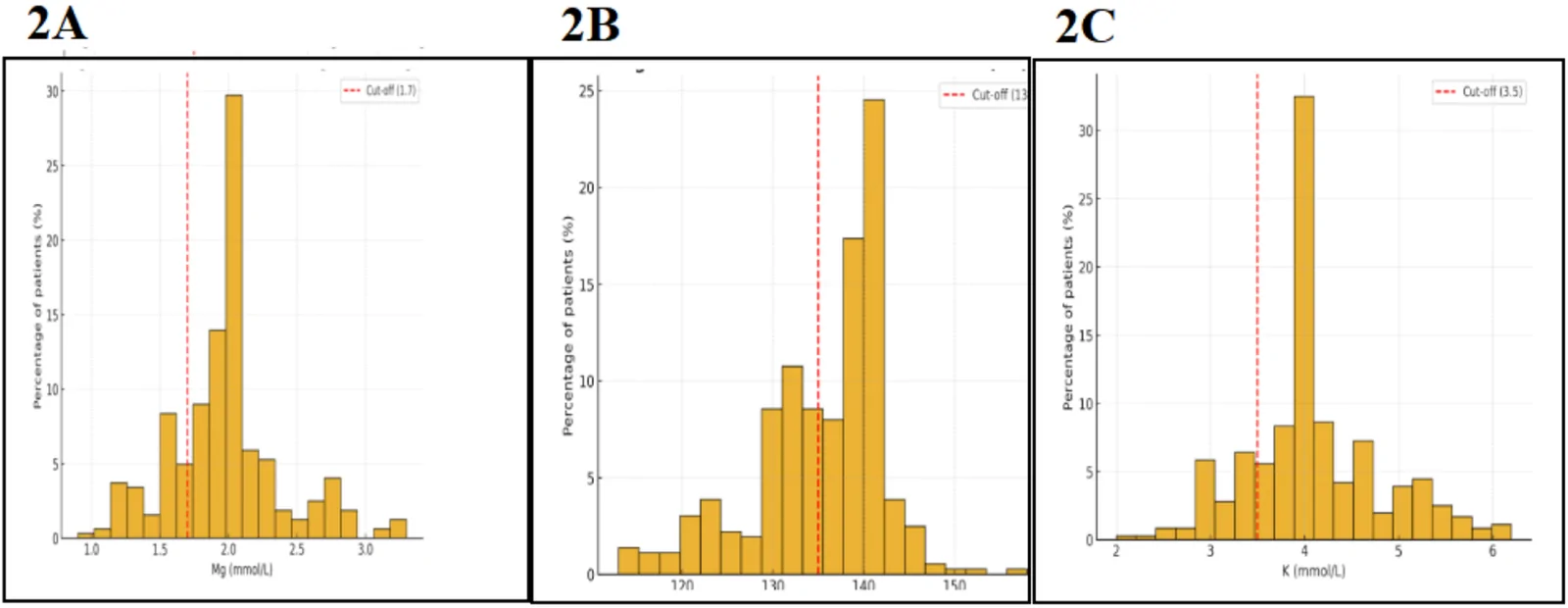
(A) Serum sodium distribution with hyponatremia cut-off (<135 mmol/L). (B) Serum potassium distribution with hypokalemia cut-off (<3.5 mmol/L). (C) Serum magnesium distribution with hypomagnesemia cut-off (<1.7 mmol/L).

**Figure 3.**
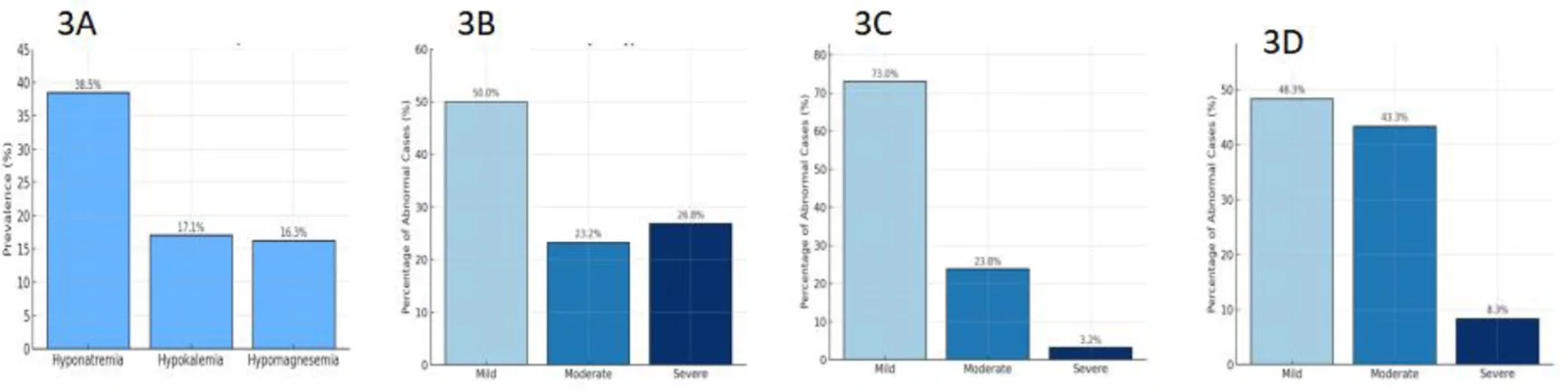
(A) Prevalence of electrolyte disturbances. Hyponatremia was most frequent (38.5%), followed by hypokalemia (17.1%) and hypomagnesen1ia (16.3%), indkating sodium imbalance as the predominant abnormality in this cohort. (B) Severity of hyponatre1nia. Mild (50.0%), moderate (23.2o/o), and severe (26.8%) reductions in sodium levels were observed, demonstrating that sodium imbalance frequently reached clinica!Jy significant levels. (C) Severity of hypokalemia. Mild cases predominated (73.0%), with fewer moderate (23.8%) and severe (3.2%) forms, suggesting mainly diuretic-related potassium loss. (D) Severity of hypomagnesemia. Mild (48.3%) and moderate (43.3o/o) deficiencies were most common, while severe hypomagnesemia(8.3%) was relatively infrequent.

Among patients with EAs, hyponatremia was the most clinically severe, with 26.8% showing a severe reduction in sodium levels. Hypokalemia was mostly mild (73.0%), while hypomagnesemia exhibited a balanced pattern between mild (48.3%) and moderate (43.3%) severities. These trends indicate that while potassium and magnesium derangements are generally moderate in this cohort, sodium imbalance often reaches critical levels, reflecting advanced or diuretic-driven HF (Fig. 3A–D).

### Factors associated with disturbances

Associations between clinical predictors and electrolyte disturbances were assessed using Chi-square tests (Table 3). Most predictors did not show statistically significant associations with hypokalemia or hyponatremia. However, calcium-channel blocker use showed a significant association with hypomagnesemia (*P* = 0.006). For some predictors (Lasix, digoxin, and cholesterol), statistical testing was not estimable because of sparse contingency table cells; therefore, *P*-values are reported as NA. No consistent statistically significant associations were observed between most clinical predictors or comorbidities and electrolyte disturbances.

**Table 3:** Associations (Chi-square tests).

Disturbance	Predictor	*P*-value	Significant (*P*<0.05)
Hypokalemia	CCB	0.148	No
Hypokalemia	DM	0.238	No
Hypokalemia	HTN	0.256	No
Hypokalemia	ACEi/ARB	0.510	No
Hypokalemia	KHAT	0.740	No
Hypokalemia	SMOKING	1.000	No
Hypokalemia	Lasix	NA	No
Hypokalemia	Digoxin	NA	No
Hypokalemia	Cholesterol	NA	No
Hypomagnesemia	CCB	0.006	Yes
Hypomagnesemia	Smoking	0.230	No
Hypomagnesemia	DM	0.687	No
Hypomagnesemia	KHAT	0.693	No
Hypomagnesemia	HTN	0.886	No
Hypomagnesemia	ACEi/ARB	0.968	No
Hypomagnesemia	Lasix	NA	No
Hypomagnesemia	Digoxin	NA	No
Hypomagnesemia	Cholestrol	NA	No
Hyponatremia	ACEi/ARB	0.273	No
Hyponatremia	DM	0.347	No
Hyponatremia	CCB	0.430	No
Hyponatremia	HTN	0.698	No
Hyponatremia	Smoking	0.844	No
Hyponatremia	Khat	0.998	No
Hyponatremia	Lasix	NA	No
Hyponatremia	Digoxin	NA	No
Hyponatremia	Cholesterol	NA	No

NA indicates that the *P*-value was not estimable due to sparse or incomplete data in contingency table cells, preventing reliable Chi-square calculation.

### Electrolytes, age, and cardiac function

Pearson correlations between electrolytes and age or ejection fraction were small in magnitude (all |*r*| ≤ 0.14), indicating no linear relationships of practical importance.

## Discussion

The prevalence of electrolyte disturbances among HF patients varies across different healthcare settings and populations^[^9^]^.

In this Somali cohort, hyponatremia was the most common EA, affecting approximately 38% of patients, followed by hypokalemia and hypomagnesemia. This finding is consistent with previous studies from African populations reporting hyponatremia in approximately 30–45% of hospitalized patients with HF. Although the present study did not evaluate clinical outcomes such as mortality or length of hospital stay, and therefore no conclusions regarding these outcomes can be drawn from our data, the high frequency of hyponatremia observed in this Somali cohort may reflect a predominance of volume-overloaded and late-presenting patients, a pattern that has also been reported in other resource-limited African healthcare settings^[^11–13^]^.

Such high prevalence rates are often attributed to late presentation, advanced disease stages, and extensive use of loop diuretics. Hyponatremia in HF is primarily driven by the activation of neurohormonal pathways, including the renin–angiotensin–aldosterone system and vasopressin secretion, which promote water retention and dilutional sodium loss^[^9^]^. Hyponatremia has also been recognized as an important prognostic marker associated with increased mortality and longer hospital stays among HF patients^[^14^]^.

In the present study, hypokalemia was observed in approximately one-fifth of participants. This finding is consistent with studies conducted in East Africa that have linked potassium depletion to loop-diuretic therapy and limited routine electrolyte monitoring^[^15,16^]^.

Magnesium deficiency was also identified in a considerable proportion of patients. Hypomagnesemia may exacerbate arrhythmias and worsen potassium depletion, making it a clinically relevant, yet frequently underdiagnosed, electrolyte disturbance in HF^[^17^]^.

Overall, the electrolyte disturbance pattern observed in this study reflects broader trends reported across sub-Saharan Africa, where delayed presentation, limited laboratory capacity, and reliance on diuretic therapy contribute to metabolic instability in patients with HF^[^18,19^]^. In contrast, studies conducted in high-income countries benefit from more comprehensive biochemical monitoring and guideline-directed therapy, allowing earlier detection and correction of EAs^[^20^]^. These findings highlight the importance of improving laboratory monitoring and strengthening clinical protocols for electrolyte management in resource-limited healthcare systems. While previous studies have demonstrated the prognostic significance of hyponatremia, the present study focused primarily on the prevalence of EAs and did not assess their impact on mortality or other clinical outcomes.

The increasing use of artificial intelligence tools in medical research and manuscript preparation has generated growing discussion regarding transparency, reliability, and ethical considerations. Recent analyses of large language models, including ChatGPT, highlight their potential to support scientific writing, literature synthesis, and language editing in biomedical research, while emphasizing the need for responsible use and clear disclosure of AI assistance^[^21^]^.

These technologies may be particularly valuable for researchers in low-resource settings by facilitating academic writing and improving the clarity of scientific communication. However, concerns remain regarding accuracy, hallucinated references, and potential biases generated by AI systems. Therefore, when such tools are used in manuscript preparation, authors should carefully verify all scientific content and provide transparent reporting in accordance with emerging guidelines for AI-assisted research.

## Limitations

Several limitations should be considered when interpreting the findings of this study. First, the retrospective design limits the ability to establish causal relationships between clinical variables and electrolyte disturbances. Second, incomplete laboratory records may have led to an underestimation of the true prevalence of EAs.

Additionally, magnesium testing was not consistently performed, and other electrolytes, such as chloride and calcium, were not routinely measured due to laboratory limitations. Important confounding variables, such as diuretic dosage, renal function markers, and nutritional status, were also not consistently available in the medical records.

Finally, the single-center design limits the generalizability of the findings to the broader Somali population.

## Conclusion

Electrolyte disturbances are highly prevalent among Somali patients hospitalized with HF, with hyponatremia representing the most frequent abnormality. The findings underscore the need for routine electrolyte monitoring and improved laboratory capacity in resource-limited healthcare settings.

Strengthening diagnostic infrastructure and implementing standardized monitoring protocols may help reduce preventable complications and improve outcomes for HF patients in Somalia.

## Data Availability

The data for this study were obtained from the hospital’s FONET electronic system and are available from the corresponding author upon reasonable request.

## Research registration unique identifying number (UIN)

Not applicable (retrospective study).
